# Farm resilience in the face of the unexpected: lessons from the COVID-19 pandemic

**DOI:** 10.1007/s10460-020-10053-5

**Published:** 2020-05-14

**Authors:** Ika Darnhofer

**Affiliations:** University of Natural Resources and Life Sciences, Vienna, Austria

The COVID-19 pandemic draws attention to many things, including our global connectedness, the role of ‘big government’, marginalisation, solidarity, and the ‘unsung heroes’ who keep essential systems running. For all of us, it tests our resilience, our ability to cope with turbulence, with the unpredictable developments in the wake of the extraordinary measures taken by governments to ‘flatten the curve’.

In Austria, in mid-March 2020 radical measures were implemented that affected almost all aspects of daily life. Although the supermarket shelves were well stocked with food, Austrians turned their attention to local farms, and farmers responded quickly. Like never before, on-line platforms were used to build new relations, connecting farmers and citizens; farmers engaged in direct marketing offered to deliver orders, as farmer markets were closed; farmers asked for support as farm workers from Eastern Europe were not allowed to enter the country, and citizens responded by volunteering to help on the fields. These are a few circumstantial observations, but they indicate how disruptive change also opens up new possibilities, and how quickly (some) farmers can adapt, demonstrating their resilience.

Studies of farm resilience focus on what enables farms to adapt to changing conditions, both on- and off-farm. They have shown that farm resilience may be influenced by available resources, workload, knowledge, power in the agro-food system, and broader societal structures. Many studies take a structuralist approach to farm resilience. They often strive to identify a ‘type’ of farm that ‘is’ resilient. So far, this has remained elusive: farms that enact resilience are rather diverse, as are the combination of challenges they face.

Taking a relational approach to farm resilience allows for different insights and may be better suited to address the unpredictable dynamics farmers have to cope with. It shifts attention from structures to relations—which may be material, social, mental—and how these are constructed, maintained, broken, adapted. Indeed, farm resilience may draw less on structural factors, and more on creativity, mental agility, possibilities explored, options kept open, honing diverse skills, bricolage, resources that can be repurposed and reallocated. In a world that is assumed predictable and is driven by efficiency, creativity and exploring possibilities may seem like daydreaming and a waste of time. Honing skills that are not currently needed or keeping unused resources may seem unproductive, but it may well be crucial to build resilience.

The question guiding research on farm resilience thus shifts from what ‘is’, to what enables an open ‘becoming’. Indeed, while crop production and animal rearing are constrained by bio-physical processes, farming might be even more constrained by what is thinkable. Yet, if there is one thing that the current COVID-19 pandemic has shown, it is that much of what was unthinkable may suddenly become a reality.

For farms, the current crisis might well highlight that a strategy which focuses on specialisation, on optimizing processes, on increasing efficiency, and on reducing production costs has its limits in a VUCA world, i.e. one that is volatile, uncertain, complex, ambiguous. Indeed, this strategy tends to reduce both the number and the diversity of relations to those few that are ‘optimal’ under specific conditions, with the implicit assumption that these conditions are controllable and will remain broadly the same. But they do not. Change is not just marginal and gradual. Surprises are inevitable. Resilience thinking teaches us to ‘expect the unexpected’.

Clearly, the current emergency measures are not here to stay. But maybe we should not rush to get back to ‘normal’. Maybe now is a good time to think what kind of agro-food system we want, and how we can transition to it. Indeed, even if the neoliberal rhetoric emphasizes that ‘there is no alternative’, the global COVID-19 pandemic clearly shows that policy makers and citizens can implement radical change. May be we should act just as decisively to address the climate crisis and the various ills in our agro-food system. May be resilience thinking can help guide this transition, as it points to the systemic benefits of encouraging diversity between and within farms; strengthening relations between farms and their local agroecosystem; and making social relations in farming more just and fair.

